# Predictive Factor for COVID-19 Worsening: Insights for High-Sensitivity Troponin and D-Dimer and Correlation With Right Ventricular Afterload

**DOI:** 10.3389/fmed.2020.586307

**Published:** 2020-11-12

**Authors:** Guillaume Goudot, Richard Chocron, Jean-Loup Augy, Nicolas Gendron, Lina Khider, Benjamin Debuc, Nadia Aissaoui, Nicolas Peron, Caroline Hauw-Berlemont, Benoit Vedie, Charles Cheng, Nassim Mohamedi, Daphné Krzisch, Aurélien Philippe, Tania Puscas, Bertrand Hermann, Julie Brichet, Philippe Juvin, Benjamin Planquette, Emmanuel Messas, Hélène Pere, David Veyer, Pascale Gaussem, Olivier Sanchez, Jean-Luc Diehl, Tristan Mirault, David M. Smadja

**Affiliations:** ^1^Vascular Medicine Department and Biosurgical Research Lab (Carpentier Foundation), AP-HP, Georges Pompidou European Hospital, Université de Paris, Paris, France; ^2^PARCC, INSERM, Université de Paris, Paris, France; ^3^Emergency Department, AP-HP, Georges Pompidou European Hospital, Université de Paris, Paris, France; ^4^Intensive Care Unit, AP-HP, Georges Pompidou European Hospital, Université de Paris, Paris, France; ^5^Innovative Therapies in Haemostasis, INSERM, Université de Paris, Paris, France; ^6^Haematology Department and Biosurgical Research Lab (Carpentier Foundation), AP-HP, Georges Pompidou European Hospital, Université de Paris, Paris, France; ^7^Plastic Surgery Department, AP-HP, Georges Pompidou European Hospital, Université de Paris, Paris, France; ^8^Biochemistry Department, AP-HP, Georges Pompidou European Hospital, Université de Paris, Paris, France; ^9^Haematology Department, AP-HP, Georges Pompidou European Hospital, Université de Paris, Paris, France; ^10^Respiratory Medicine Department and Biosurgical Research Lab (Carpentier Foundation), AP-HP, Georges Pompidou European Hospital, Université de Paris, Paris, France; ^11^Virology Department, AP-HP, Georges Pompidou European Hospital, Université de Paris, Paris, France; ^12^Centre de Recherche des Cordeliers, Functional Genomics of Solid Tumors, INSERM, Université de Paris, Paris, France; ^13^Intensive Care Unit and Biosurgical Research Lab (Carpentier Foundation), AP-HP, Georges Pompidou European Hospital, Université de Paris, Paris, France

**Keywords:** COVID-19, troponin, D-dimer (DD), echocardiograghy, thrombosis, right ventricle

## Abstract

**Background:** Coronavirus disease 2019 (COVID-19) has been associated with cardiovascular complications and coagulation disorders.

**Objectives:** To explore clinical and biological parameters of COVID-19 patients with hospitalization criteria that could predict referral to intensive care unit (ICU).

**Methods:** Analyzing the clinical and biological profiles of COVID-19 patients at admission.

**Results:** Among 99 consecutive patients that fulfilled criteria for hospitalization, 48 were hospitalized in the medicine department, 21 were first admitted to the medicine ward department and referred later to ICU, and 30 were directly admitted to ICU from the emergency department. At admission, patients requiring ICU were more likely to have lymphopenia, decreased SpO_2_, a D-dimer level above 1,000 ng/mL, and a higher high-sensitivity cardiac troponin (Hs-cTnI) level. A receiver operating characteristic curve analysis identified Hs-cTnI above 9.75 pg/mL as the best predictive criteria for ICU referral [area under the curve (AUC), 86.4; 95% CI, 76.6–96.2]. This cutoff for Hs-cTnI was confirmed in univariate [odds ratio (OR), 22.8; 95% CI, 6.0–116.2] and multivariate analysis after adjustment for D-dimer level (adjusted OR, 20.85; 95% CI, 4.76–128.4). Transthoracic echocardiography parameters subsequently measured in 72 patients showed an increased right ventricular (RV) afterload correlated with Hs-cTnI (*r* = 0.42, *p* = 0.010) and D-dimer (*r* = 0.18, *p* = 0.047).

**Conclusion:** Hs-cTnI appears to be the best relevant predictive factor for referring COVID-19 patients to ICU. This result associated with the correlation of D-dimer with RV dilatation probably reflects a myocardial injury due to an increased RV wall tension. This reinforces the hypothesis of a COVID-19-associated microvascular thrombosis inducing a higher RV afterload.

## 1. What is known about this topic?

COVID-19 is associated with cardiovascular complications and coagulation disorders.
Predictive markers of severity and intensive care unit (ICU) referral are required upon hospital admission.

## 2. What does this paper add?

D-Dimer and high sensitivity cardiac troponin (Hs-cTnI) at hospital admission are prognosis biomarkers for ICU referral.
Hs-cTnI appears the best relevant predictive factor for ICU referral in COVID-19 patients.
D-Dimer and Hs-cTnI elevation are correlated with the increase of right ventricular afterload observed in COVID-19.

## Introduction

Severe acute respiratory syndrome coronavirus 2 (SARS-CoV-2) infection can be asymptomatic or lead to the coronavirus disease 2019 (COVID-19), which has not only a very large pattern of respiratory manifestations but also other non-specific symptoms including fever, headache, hemoptysis, nausea, vomiting, and diarrhea also previously described in other coronavirus infections (1, 2). In terms of respiratory symptoms, COVID-19 is characterized by a large spectrum of infectious signs from dry cough and pulmonary edema to acute respiratory distress syndrome (ARDS), requiring hospitalization in intensive care unit (ICU) and leading to death in the most severe cases (2). While patients with cardiovascular comorbidities are described to have the higher mortality rate in published data (3), it is however challenging to provide a risk stratification of disease progression at admission for COVID-19 patients. Respiratory condition can worsen rapidly and is currently difficult to predict. From the very first consultation of the patient, biological markers could help to predict COVID-19 systemic consequences that would require ICU referral.

Several biomarkers of COVID-19 have been associated with the disease severity and progression. COVID-19-associated coagulopathy was found more frequent in fatal COVID-19 cases, and a high D-dimer level has been associated with poor prognosis and in-hospital mortality (4–6). The hypothesis of the presence of microthrombi in lungs and kidneys was suggested after autopsy case series (7–9). In COVID-19, microvascular thrombosis is observed during an inflammatory storm and could participate in damaging capillary endothelium and disrupting the thrombo-protective state of endothelial cells (10–12). SARS-CoV-2 has been shown to infect blood vessels and induce vascular damage *in vitro* and *in vivo* (13–15). SARS-CoV-2 can infect cells via the angiotensin-converting enzyme 2 (ACE2) receptor, which is ubiquitous but largely expressed in endothelial cells (16). Endotheliitis could be at the origin of impaired microcirculatory function affecting the lungs and kidneys (15, 17, 18). Furthermore, we previously described the endothelial lesion in patients with hospitalization criteria as a marker of COVID-19 severity at hospital admission (19). Acute myocardial injuries were also largely reported, such as numerous acute coronary syndromes or myocarditis (20). Cardiac troponin is increased in the case of community-acquired pneumonia, in the context of myocardial oxygen supply–demand mismatch and supply (21), and seems to correlate with the severity of respiratory impairment. Yet, few data are currently available on troponin level at the time of COVID-19 diagnosis (20, 22–24). Mechanisms of myocardial and tissue injury as well as coagulopathy associated with COVID-19 could be linked to the cytokine storm (25).

In this study, we aimed at identifying, in COVID-19 patients with criteria for hospitalization, biological and clinical markers at admission that could predict future referral in ICU and help anticipating a worsening of the patient's condition. We also investigated correlations between biological markers and cardiac function, assessed by ultrasound.

## Methods

### Study Design and Population

This study is an observational cohort study conducted at the Georges Pompidou European Hospital in Paris, France. We prospectively included consecutive patients with confirmed SARS-CoV-2 infection. Inclusion criteria were patients over 18 years of age, with COVID-19, who consulted the emergency department with hospitalization criteria. Primary endpoint was ICU transfer, according to the usual criteria of ICU requirement, described in Table 1, and was kept unchanged throughout the study. All patients were confirmed with SARS-CoV-2 infection by nasopharyngeal swabs. For each patient included, clinical evaluation and computed tomography (CT) scan and biological evaluation were performed. For all patients, baseline characteristics (demographics, treatment, clinical examination, cardiovascular risk factors, and body mass index) and biological data were retrieved from the medical records using a standardized data collection.

**Table 1:** Criteria to admit COVID-19 patients to ICU.

**ICU referral criteria for COVID-19 patients**
Respiratory failure requiring mechanical ventilation at 6–8 L/min of oxygen to maintain SpO_2_ >90–92%
and/or signs of respiratory distress (≧30 breaths/min), thoraco-abdominal swaying, inspiratory depression of the suprasternal trough
and/or other associated failure (s): loss of consciousness with Glasgow Coma Scale <12; systolic arterial pressure <90 mmHg, signs of peripheral hypoperfusion

*ICU, intensive care unit; SpO_2_, pulse oximetric saturation*.

### Routine Blood Examinations

All samples were collected in ethylenediaminetetraacetic acid (EDTA), sodium heparin, and 0.129 M trisodium citrate tubes (9NC BD Vacutainer^©^). Routine lab tests included complete blood count, creatinine, C-reactive protein (CRP), and high-sensitivity cardiac troponin (Hs-cTnI, Beckman) on a DXI analyzer (26, 27). Coagulation tests were prothrombin time (PT) ratio, fibrinogen, and soluble fibrin monomer level (STA-Liatest FM® Diagnostica Stago^©^) explored on a STA-R® Max coagulometer (Stago^©^) as previously described (28). D-Dimer concentrations were determined using the Vidas D-Dimer assay (BioMérieux^©^) according to the manufacturer's instructions.

### Transthoracic Cardiac Ultrasound and ICU Respiratory Parameters Evaluation

Cardiac ultrasound was performed on 72 additional consecutive COVID-19 patients, in the medicine department (*n* = 32) and ICU (*n* = 40). Transthoracic echocardiography (TTE) was performed using commercially available equipment CX50®, S5-1 probe (1–5 MHz; 80 elements) (Philips Medical Systems^©^, Andover, Massachusetts), according to the guidelines of the American Society of Echocardiography, during the first 24 h after hospital admission (29). At the time of echocardiographic examination, heart rate (HR), systolic, diastolic, and mean blood pressure were recorded. The examination included standard parasternal view to assess the size of the left ventricle (LV) [LV end diastolic diameter, (LVEDD); LV end systolic diameter (LVESD)]. LV ejection fraction was determined using the Simpson's method. Velocity time integral (VTI, cm) of the LV outflow tract and cardiac output were measured on an apical five-chamber view. Using the apical four-chamber view, mitral inflow was recorded by pulse-wave Doppler. We assessed the early diastole (E, cm/s) and the atrial contraction (A, cm/s). Early diastole (e′, cm/s) velocity of the lateral mitral annulus was measured by tissue Doppler imaging. We then calculated the E/A and E/e′ ratios. Using the same view, we carried out an evaluation of the right ventricle (RV): tricuspid annular plane systolic excursion (TAPSE) with TM mode, S wave velocity with tissue Doppler imaging, peak systolic tricuspid insufficiency velocity, and ratio of basal diameters of the right and left ventricle (RV/LV). The trans-tricuspid pressure difference was estimated from the peak velocity of tricuspid regurgitant jet (TR Vmax) by applying the simplified Bernoulli equation. The systolic pulmonary arterial pressure (sPAP) was then derived by adding estimated right atrium pressure based on the inspiratory changes in the dimension of inferior vena cava (30).

We also collected respiratory characteristics [FiO_2_, positive end-expiratory pressure (PEEP), plateau pressure (Pplat), respiratory system compliance] and hemodynamic parameters [mean arterial pressure (MAP), HR, catecholamine dose] at the time of TTE assessment. The blood gas samples were collected just before the TTE.

### Statistical Analysis

Continuous data were expressed as median [interquartile range (IQR)] and categorical data as proportion. Patients were compared according to patients' care pathway divided into three groups: patient hospitalized in the medical department, patients hospitalized in the medical department and then referred to ICU following respiratory worsening, and patients directly admitted to the ICU. In this univariate analysis, continuous variables were compared using Kruskal–Wallis test, and categorical variables were compared using Cochran–Armitage test for trend (multiple group). Patients were also compared according to the Hs-cTnI at admission. In this univariate analysis, we determined the differences in median using the unpaired *t*-test (Mann–Whitney *U* test) for continuous variable, and differences in proportions were assessed with the chi-square test or Fisher's exact test if necessary. We generated receiver operating characteristics (ROC) curves with four different regression models that included variables with significant difference in the univariate analysis (31, 32). Models included (i) plasma level of D-dimer (over a cutoff value of 1,000 ng/mL only), (ii) the Hs-cTnI only, (iii) the Hs-cTnI adjusted on gender, the presence of pneumonia on CT scan and the plasma level of D-dimer (over a cutoff value of 1,000 ng/mL), and (iv) the gender, the presence of pneumonia on CT scan, and the plasma level of D-dimer (over a cutoff value of 1,000 ng/mL). The four models helped assess the extent to which the level of D-dimer and Hs-cTnI influenced the predictability of hospitalization in ICU. We calculated the area under the curve (AUC) for the different logistic regression model (32, 33). We used logistic regression to determine whether the level of D-dimer (as a categorical dependent variable dichotomized according to the cutoff of 1,000 ng/mL) and the level of Hs-cTnI (as a categorical dependent variable dichotomized according to the cutoff of 9.75 pg/mL) were associated with the ICU referral (34, 35). The model included only these two variables (D-dimer and Hs-cTnI), and to take into account any potential interaction, we performed the model with and without interaction term. The correlation between biological parameters (Hs-cTnI and D-dimer) at hospital admission and ultrasound characteristics of patients who were hospitalized in ICU with invasive mechanical ventilation was assessed using the Kendall coefficient correlation test. All analyses were two-sided and a *p* < 0.05 was considered statistically significant. Statistical analysis was performed using R studio software (R^©^ Development Core Team, 2019).

## Results

### D-Dimer and Hs-cTnI Levels at Hospital Admission Are Discriminant Biomarkers to Predict ICU Referral

The cohort study was composed of 99 consecutive patients who presented to the emergency department and were diagnosed with COVID-19 in March and April 2020. They were divided into three groups: patients hospitalized in the medical department (*n* = 48), patients first hospitalized in the medicine department (mean of 3.0 ± 1.4 days) then referred to ICU due to respiratory degradation (*n* = 21), and patients admitted to ICU after admission to the emergency department (*n* = 30).

These three groups were strictly comparable in terms of age, body mass index, cardiovascular risk factors, treatments, and time from illness onset to hospitalization (Table 2). However, COVID-19 patients directly admitted to ICU had more often history of coronary heart disease and were more likely to have dyspnea at admission (*p* < 0.001), decreased SpO_2_ (*p* < 0.001), pneumonia on the CT scan (*p* = 0.002), ARDS (*p* < 0.001), and increased respiratory rate–breath per minute (*p* < 0.001). In terms of biological features, patients directly requiring ICU admission and those referred to ICU after conventional hospitalization had a significantly higher white blood cell count and granulocytes count (respectively, *p* = 0.03 and 0.003) with more severe lymphopenia and monocytopenia (respectively, *p* = 0.002 and 0.005) than patients hospitalized in the medicine department. Regarding coagulation disorders, 70% of COVID-19 patients admitted to ICU had D-dimer level above 1,000 ng/mL at hospital admission (*p* = 0.0013). The PT ratio was significantly different between groups; however, it remained within normal range. In the whole COVID-19 population, fibrin monomers were negative and associated with hyperfibrinogenemia and without thrombocytopenia. These results were not in favor of a COVID-19-associated disseminated intravascular coagulation (DIC). Lastly, COVID-19 patients admitted to ICU had a significantly higher CRP level (*p* < 0.001), plasma creatinine (*p* = 0.002), and Hs-cTnI (*p* < 0.001). No correlation was found between Hs-cTnI and D-dimer (*p* = 0.82), Hs-cTnI and creatinine (*p* = 0.27), or Hs-cTnI and SpO_2_ (*p* = 0.13), while a significant association was found between Hs-cTnI and CRP (*p* = 0.001).

**Table 2:** Demographic and clinical characteristics of patients at admission according to the addressed department (medical or ICU).

	**Medicine patients (*n* = 48)**	**Medicine then ICU patients (*n* = 21)**	**ICU patients (*n* = 30)**	***p-*Value**
Male sex—*n* (%)	31 (64.6)	17 (81.0)	26 (86.7)	0.070
Age—years, median (IQR)	62.5 (50.8–80.0)	67.0 (55.0–75.0)	60.0 (55.0–69.8)	0.764
BMI—kg/m^2^, median (IQR)	26.3 (24.7, 29.3)	26.7 (24.9, 28.1)	27.3 (25.0–30.5)	0.810
Time from illness onset to hospital admission—days	4.5 (3.0–7.0)	7.0 (4.0–8.0)	7.0 (4.0–9.0)	0.073
**CV risk factors**, ***n*** **(%)**				
Hypertension	19 (39.6)	11 (52.4)	16 (53.3)	0.624
Dyslipidemia	11 (22.9)	5 (3.8)	9 (30.0)	0.771
Diabetes	6 (12.5)	8 (38.1)	9 (30.0)	0.062
Sedentary lifestyle	4 (8.3)	0 (0.0)	2 (6.7)	0.715
Chronic kidney disease	4 (8.3)	4 (19.0)	3 (10.0)	0.416
**Medical history**, ***n*** **(%)**				
Cancer	4 (8.3)	1 (4.8)	1 (3.3)	0.641
Coronary heart disease	4 (8.3)	1 (4.8)	5 (16.7)	0.002
Stroke	3 (6.2)	2 (9.5)	2 (6.7)	0.883
**Treatments**, ***n*** **(%)**				
Statins	11 (22.9)	5 (23.8)	9 (30.0)	0.771
Oral antidiabetic agents	5 (10.4)	6 (28.6)	8 (26.7)	0.098
Insulin	2 (4.2)	4 (19.0)	3 (10.0)	0.138
β-blocker	5 (10.4)	3 (14.3)	5 (16.7)	0.718
Calcium channel blockers	8 (16.7)	6 (28.6)	5 (16.7)	0.470
ACEi or ARBs	13 (27.1)	6 (28.6)	12 (40.0)	0.466
ARBs	6 (12.5)	3 (14.3)	5 (16.7)	0.864
Diuretics	4 (8.3)	4 (19.0)	4 (13.3)	0.442
Central acting agent	1 (2.1)	0 (0.0)	0 (0.0)	0.585
**Clinical features**, ***n*** **(%)**				
Fever	44 (91.7)	20 (95.2)	28 (93.3)	0.863
Headache	10 (20.8)	8 (38.1)	15 (50.0)	0.089
Cough	33 (68.8)	19 (90.5)	25 (83.3)	0.268
Productive cough	6 (12.5)	1 (4.8)	2 (6.7)	0.505
Dyspnea	19 (39.6)	15 (71.4)	28 (93.3)	<0.001
Myalgia	14 (29.2)	6 (28.6)	12 (40.0)	0.559
Diarrhea	3 (6.2)	7 (33.3)	4 (13.3)	0.045
Pneumonia	32 (66.7)	19 (90.5)	29 (96.7)	0.002
ARDS	0 (0.0)	2 (9.5)	11 (36.7)	<0.001
SpO_2_–%, median (IQR)	95.0 (92.5–96.0)	92.0 (90.0–96.0)	89.0 (84.0–92.0)	<0.001
Respiratory rate—breathes per min, median (IQR)	18.0 (16.0–20.0)	20.0 (16.0–25.0)	23.0 (21.0–32.0)	0.001
Pulse—beats per min, median (IQR)	87.0 (76.5–99.0)	88.0 (80.0–98.0)	97.0 (87.0–110.0)	0.060
**Biological parameters**, ***n*** **(%)**				
White blood cells—× 10^9^/L, median (IQR)	5.85 (4.52–7.03)	4.60 (4.20–6.90)	7.20 (5.10–11.10)	0.034
Hemoglobin—g/L, median (IQR)	130.5 (111.5–148.0)	140.0 (129.0–151.0)	130.0 (119.0–140.0)	0.213
Platelet count—× 10^9^/L, median (IQR)	171.5 (149.8–228.0)	147.0 (117.0–197.0)	179.0 (138.0, 247.0)	0.042
Polynuclear neutrophils—× 10^9^/L, median (IQR)	3.92 (2.94–5.36)	3.74 (2.55–5.90)	6.14 (3.99–10.00)	0.003
Lymphocytes—× 10^9^/L, median (IQR)	0.97 (0.76–1.35)	0.74 (0.63–1.01)	0.60 (0.44–0.95)	0.002
Monocytes—× 10^9^/L, median (IQR)	0.48 (0.35–0.66)	0.34 (0.24–0.44)	0.34 (0.23–0.53)	0.005
CRP—mg/L, median (IQR)	64.8 (14.3–100.4)	104.0 (57.1–162.0)	164.0 (105.5–209.8)	<0.001
Plasma creatinine—μmol/L, median (IQR)	72.0 (60.0, 89.0)	89.0 (80.0–119.0)	101.0 (75.5–179.3)	0.002
Hs-TNI—pg/mL, median (IQR)	5.6 (4.3–11.3)	20.0 (10.5–35.5)	26.0 (18.0–95.0)	<0.001
PT ratio, median (IQR)	0.96 (0.91–1.03)	0.94 (0.91–1.00)	0.86 (0.77–0.96)	0.009
Fibrinogen—g/L, median (IQR)	5.1 (4.7–5.8)	5.7 (5.6–6.5)	6.5 95.8–7.3)	<0.001
D-dimer ≥1,000 ng/mL—***n*** (%)	15 (31.2)	11 (52.4)	21 (70.0)	0.013
D-dimer—ng/mL, median (IQR)	840 (570–1,462)	1,455 (630–2,003)	1,358 (957–2,122)	0.060
Fibrin monomers—μg/mL, median (IQR)	<7.0 (<7.0–<7.0)	<7.0 (<7.0–<7.0)	<7.0 (<7.0–<7.0)	0.876

*ICU, intensive care unit; BMI, body mass index; CV, cardiovascular; ACEi, angiotensin conversion enzyme inhibitor; ARB-2, antagonist of angiotensin 2 receptor blocker; SpO_2_, pulse oximetric saturation; ARDS, acute respiratory distress syndrome; IQR, interquartile range; CRP for C-reactive protein; Hs-TnI for ultrasensitive troponin I; PT, prothrombin time. p trend between three groups*.

### Hs-cTnI Level at Entrance Is the Most Relevant Biomarker to Predict ICU Referral

**Figure 1:**
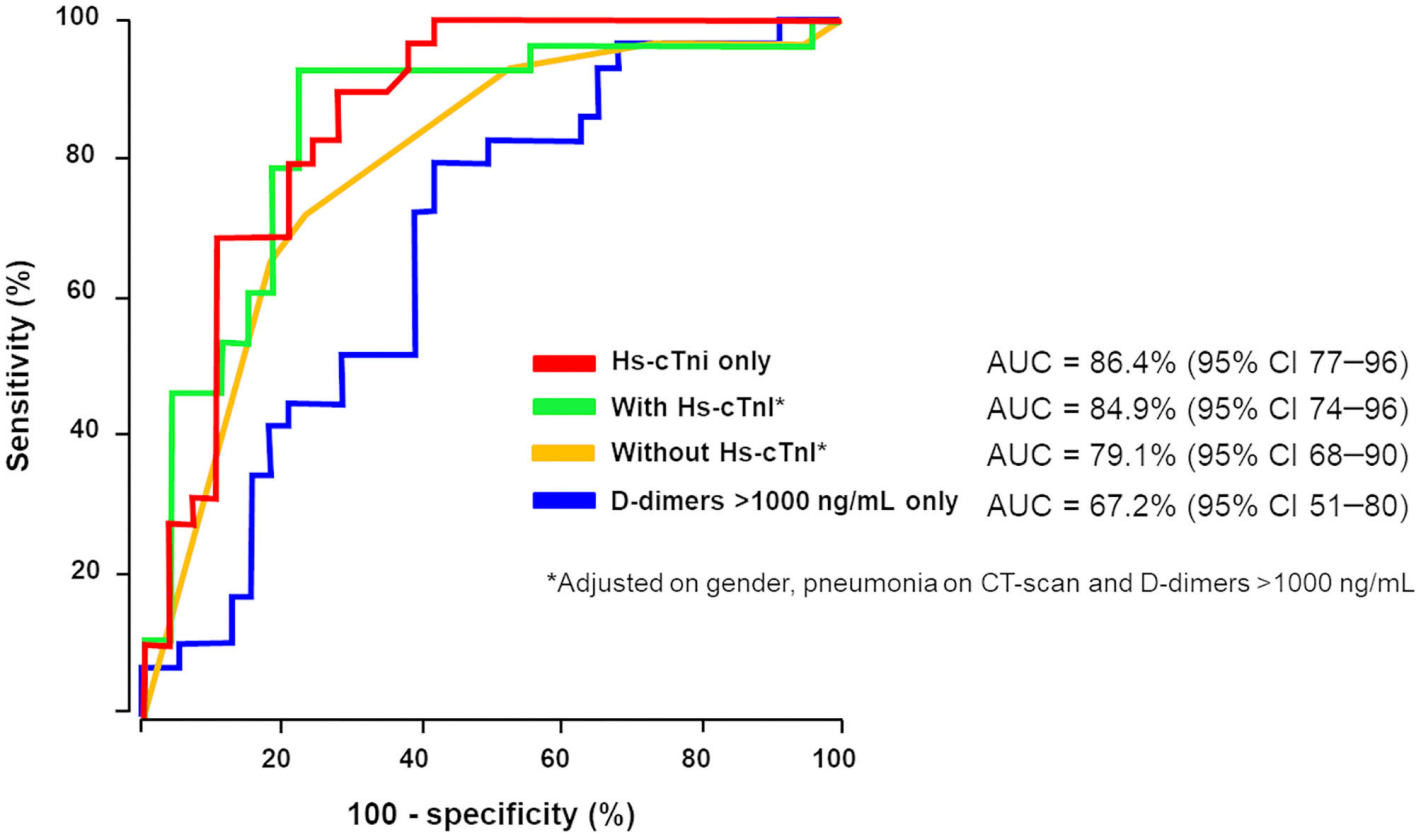
D-Dimer and high-sensitivity cardiac troponin (Hs-cTnI) involvement in intensive care unit (ICU) referral. We used different D-dimer level cutoffs (>1,000 ng/mL, >2,000 ng/mL, >3,000 ng/mL) as potential prognostic criteria for ICU referral. Receiver operating characteristics (ROC) curve analysis associating D-dimer above 1,000 ng/mL, gender and pneumonia at CT scan for ICU transfer (in yellow) increases area under the curve (AUC) in contrast to D-dimer ≥1,000 ng/mL alone (AUC, 79.1; 95% CI, 68–90, *p* = 0.04). Addition of Hs-cTnI to this model (in green) allowed reaching AUC of 84.9 (95% CI, 74–96, *p* = 0.03). Hs-cTnI alone was the best predictive ROC curve (in red) for ICU outcome with AUC of 86.4 (95% CI, 77–96).

In the context of coagulopathy and myocardial injury during COVID-19 and given the observation that D-dimer and Hs-cTnI levels at admission were associated with ICU admission, a ROC curve analysis was constructed using these biomarkers (Figure 1). We used different D-dimer level cutoffs (>1,000, >2,000, and >3,000 ng/mL) as potential prognostic criteria for ICU referral. For the cutoff of >1,000 ng/mL, the ROC curve (AUC, 67.2; 95% CI, 51.4–80.2) yielded a sensitivity of 72.4% (95% CI, 50.0–86.0), a specificity of 60.0% (95% CI, 43.0–75.0), a positive predictive value (PPV) of 58.6% (95% CI, 40.0–74.0), and a negative predictive value (NPV) of 74.2% (95% CI, 55.0–87.0%, Table 3). None of the other cutoffs had better prognostic values (Table 3). The D-dimer cutoff of 1,000 ng/mL as a predictive value for ICU referral was improved in a ROC curve analysis, when associated with gender and pneumonia at CT scan (AUC, 79.1; 95% CI, 68.5–89.7, *p* = 0.04). The addition of Hs-cTnI to this model allowed reaching an AUC of 84.9 (95% CI, 73.9–95.9, *p* = 0.03). Furthermore, Hs-cTnI alone was the best predictor for ICU outcome with AUC of 86.4 (95% CI, 76.6–96.2). ROC curve identified a cutoff at 9.75 pg/mL yielding a high sensitivity of 89.6% (95% CI, 71.0–97.0), a good specificity of 72.4% (95% CI, 52.0–86.0), a high PPV of 76.5% (95% CI, 58.0–88.0), and a high NPV of 87.5% (95% CI, 66.0–96.0). None of the other Hs-cTnI cutoffs currently used for acute myocardial infarction [11.6 and 19.8 pg/mL, respectively, for men and women; cutoff detection for acute myocardial injury defined as an elevated Hs-cTnI value above the 99th percentile upper reference limit (27)] had a prognostic value better than 9.75 pg/mL (Table 4).

**Table 3:** Evaluation of various D-dimer cutoffs at admission related to ICU referral.

**D-dimer involvement in ICU referral**						
**Cutoff**	**1,000 ng/mL**		**2,000 ng/mL**		**3,000 ng/mL**	
		**95% CI**		**95% CI**		**95% CI**
Sensitivity	72.4%	52–86	34.0%	18–54	10.0%	20–28
Specificity	60%	43–75	84.2%	68–93	86.3%	71.0–95
Positive predictive value	58.6%	40–74	62.5%	35–83	37.5%	10–74
Negative predictive value	74.2%	55–87	62.7%	48–75	55.9%	42–68

*ICU, intensive care unit*.

**Table 4:** Various Hs-cTnI cutoffs at admission related to ICU referral.

**Hs-cTnI involvement in ICU referral**						
	**Cutoff:** **9.75 pg/mL**		**Cutoff:** **11.6 pg/mL**		**Cutoff:** **19.8 pg/mL**	
		**95% CI**		**95% CI**		**95% CI**
Sensitivity	89.6%	71–97	82.7%	63–93	68.0%	49–84
Specificity	72.4%	52–86	75.8%	57–89	82.0%	63–93
Positive predictive value	76.5%	58–88	77.4%	58–89	80.0%	58–92
Negative predictive value	87.5%	66–96	81.5%	64–92	72.0%	54–86

*Hs-cTnI, high sensitivity cardiac troponin; ICU, intensive care unit*.

Table 5 confirms the link between Hs-cTnI and ICU referral using a logistic regression model with the cutoff of 9.75 pg/mL for Hs-cTnI (OR, 22.8; 95% CI, 6.0–116.2, *p* < 0.001). Strikingly, when adjusted to D-dimer level, the adjusted OR of 20.85 (95% CI, 4.76–128.4, *p* < 0.001) was not better. Inversely, when a logistic regression model used the cutoff of 1,000 ng/mL for D-dimer, the association between D-dimer and ICU outcome (OR, 4.02; 95% CI, 1.46–11.93, *p* = 0.009) did not remain significant when adjusted to Hs-cTnI (OR, 1.34; 95% CI, 0.3–5.9, *p* = 0.7), confirming the higher specificity of Hs-cTnI in predicting ICU referral in hospitalized patients. The addition of the interaction term between D-dimer and Hs-cTnI did not change the associations observed. To confirm the relevance of this proposed Hs-cTnI cutoff of 9.75 pg/mL, we compared clinical and biological results in the study population and found that patients with Hs-cTnI above 9.75 pg/mL were older (*p* = 0.01) and more likely to get a β-blocker prescription (*p* = 0.034), angiotensin-converting enzyme inhibitors (ACEi) or angiotensin receptor blockers (ARBs) therapy (respectively, *p* = 0.005 for ACEi or ARBs and *p* = 0.021 for ARBs alone), or a history of coronary heart disease (*p* = 0.016) (Supplementary Table 3). Lastly, we evaluated the link between Hs-cTnI and mortality among the same patients. The ROC curve identified a cutoff at 10.75 pg/mL yielding a sensitivity of 42.6% (95% CI, 29.5–56.8) and a specificity of 100% (95% CI, 80.7–100). We will confirm the relevance of an Hs-cTnI threshold of 9.75 pg/mL in a prospective study.

**Table 5:** Logistic regression model evaluating D-dimer >1,000 ng/mL and high-sensitivity troponin level in ICU referral and mortality.

**Logistic regression model with ICU referral as the outcome**				
		**OR (univariate) 95% CI**	**OR (bivariate) 95% CI**	**OR (bivariate with interaction term) 95% CI**
D-dimer—ng/mL	<1,000	–	–	–
	>1,000	4.02 (1.46–11.93, *p* = 0.009)	1.34 (0.25–5.88, *p* = 0.706)	1.41 (0.06–19.35, *p* = 0.760)
Hs-TnI—pg/mL	<9.75	–	–	–
	>9.75	22.75 (6.03–116.17, p <0.001)	20.85 (4.76–128.40, *p* < 0.001)	21.50 (3.07-271.12, *p* = 0.005)
Interaction term between Hs-TnI and D-dimer				0.84 (0.03–30.50, *p* = 0.918)
Metrics of the model	C-statistic		0.825	0.825
	AIC		57.3	59.4
**Logistic regression model with mortality as the outcome**				
D-dimer—ng/mL	<1,000	–	–	–
	>1,000	3.22 (1.17–9.94, *p* = 0.030)	1.49 (0.44–5.22, *p* = 0.521)	2.71 (0.10–75.26, *p* = 0.500)
Hs-TnI—pg/mL	<9.75	–	–	–
	>9.75	9.50 (2.44–63.36, *p* = 0.004	8.46 (1.99–59.23, *p* = 0.010)	11.87 (1.59–247.68, *p* = 0.035)
Interaction term between Hs-TnI and D-dimer				0.49 (0.01–16.54, *p* = 0.657)
Metrics of the model	C-statistic		0.730	0.725
	AIC		80.3	82

*The model included only two variables (D-dimer and Hs-cTnI) with and without interaction term*.

*Hs-cTnI, high-sensitivity cardiac troponin; ICU, intensive care unit; OR, odds ratio; C-statistic, concordance statistic; AIC, Akaike information criterion*.

### D-Dimer and Hs-cTnI Levels Are Related to Vascular Obstruction and Increased Right Ventricular Afterload on Transthoracic Cardiac Ultrasound Evaluation

We explored the cardiac function by TTE of 72 consecutive patients tested positive for COVID-19, admitted in April 2020, 32 (44%) patients in the medicine department, and 40 (56%) patients in ICU. Demographic data and respiratory function are reported in Supplementary Tables 1, 2. Regarding patients in ICU, at the time of TTE assessment, median PEEP was 13 cmH_2_O (5–16), median Pplat was 26 cmH_2_O (19–28), median respiratory system compliance was 36.30 (mL/cmH_2_O) (31.1–45.9), and FiO_2_ 50% (44–100). Regarding hemodynamic parameters median MAP was 70 mmHg (60–78), median HR was 105/min (85–116), while median epinephrine dose was 0.0 mg/h (0.0–0.9). Most patients had respiratory acidosis with median pH 7.28 (7.20–7.34), median PaCO_2_ of 53 mmHg (42–59), median PaO_2_ of 85 mmHg (68–100), and median lactate of 1.45 mmol/L (1.1–2.0).

As described in Supplementary Table 4, we found no change in the LV ultrasound parameters. No significant abnormalities in the LV geometry [LVEDD at 44.0 (41.9–50) mm or LVESD at 31.0 (26.4–37) mm], LV ejection fraction [60 (55–65)%], or LV filling pressures [E/A ratio at 0.9 (0.7–1.4)], with a median E wave at 75 (59–85.2) cm/s and E/lateral e′ ratio at 6.2 (4.9–8.3), were indeed observed. Regarding the relationship between biological parameters at hospital admission and ultrasound characteristics, Hs-cTnI showed a significant but weak correlation with the LV ejection fraction (*r* = −0.196, *p* = 0.039) and no correlation with the E/e′ ratio (*p* = 0.157). D-Dimer did not correlate with these parameters. We did not find any acute systolic dysfunction associated with COVID-19. Hs-cTnI elevation corresponded rather to a higher rise in heart disease with underlying low LVEF.

Concerning the RV, we found an initial dilatation of the RV diameter [median diameter of 37.8 (33.0–43.3) mm], with an RV/LV ratio of 0.8 (0.7–0.9), without dilatation of the LV. RV function was maintained [s′ tissue Doppler imaging (TDI) at 13.0 (11.5–16.0) cm/s; tricuspid annular plane systolic excursion (TAPSE) at 21.0 (17.8–23.6) mm]; the systolic pulmonary arterial pressure (sPAP) value remained above normal but without major elevation [31.6 (25.1–43.6) mmHg]. Regarding the ultrasound parameters of the RV, the strongest correlation was between Hs-cTnI level and sPAP (*r* = 0.42; *p* = 0.01, Table 6). Hs-cTnI was also correlated with an RV systolic dysfunction (correlation with TAPSE, *r* = −0.24; *p* = −0.007) but not with an RV dilatation (RV/LV ratio, *p* = −0.765). D-Dimer levels showed significant correlations with the same parameters, sPAP (*r* = 0.18, *p* = 0.046), TAPSE (*r* = 0.18, *p* = 0.035), and with the dilatation of the RV (*r* = 0.23, *p* = 0.012).

**Table 6:** Correlations between biological markers and echocardiographic features.

**Biomarker**	**TTE parameter**	**Correlation coefficient**	***p*-Value**
**Left ventricle parameters**			
Hs-cTnI	LVEF	−0.195	0.039
Hs-cTnI	E/e′ ratio	0.157	0.076
D-dimer	LVEF	0.027	0.770
D-dimer	E/e′ ratio	0.092	0.288
**Right ventricle parameters**			
Hs-cTnI	RV diameter	0.177	0.060
Hs-cTnI	RV/LV ratio	−0.028	0.765
Hs-cTnI	sPAP	0.425	0.010
Hs-cTnI	TR Vmax	0.380	0.010
Hs-cTnI	TAPSE	−0.236	0.007
Hs-cTnI	S wave (RV)	−0.133	0,329
D-dimer	RV diameter	0.234	0.012
D-dimer	RV/LV ratio	0.147	0.116
D-dimer	sPAP	0.178	0.047
D-dimer	TR Vmax	0.201	0.026
D-dimer	TAPSE	−0.181	0.035
D-dimer	s wave (RV)	0.161	0.251

*Hs-cTnI, high-sensitivity cardiac troponin; LVEF, left ventricular ejection fraction; RV, right ventricle; LV, left ventricle; sPAP, systolic pulmonary arterial pressure; TAPSE, tricuspid annular plane systolic excursion; S wave (RV), positive systolic wave of the right ventricle using Tissue Doppler imaging*.

## Discussion

In this prospective single-center study, we reported that Hs-cTnI level at admission was the best biomarker to predict ICU transfer and respiratory severity in COVID-19 patients. Moreover, we evidenced the D-dimer involvement in the pathophysiology of COVID-19 and the correlation with a RV afterload, which allows us to confirm pulmonary vascular obstruction as a site of coagulopathy and a source of circulating D-dimer.

D-dimer increase has been widely reported during SARS-CoV-2 infection (2, 17, 36–38). It has been first associated with sepsis-induced coagulopathy and with DIC (37, 39). However, beside a high D-dimer level, the present study evidenced a low level of fibrin monomers, a high fibrinogen level, and no signs of thrombocytopenia, allowing us to exclude a DIC at admission. D-dimer may reflect the consequences of the COVID-19-associated coagulopathy (40, 41), as it probably participates in the respiratory disease through the development of capillary microthrombosis as observed in postmortem studies (42) and attributed to a vascular thickening or vascular congestion (14, 43–45). Moreover, another pulmonary vascular issue in COVID-19 is related to a high incidence of pulmonary embolism (PE) (46, 47) whose exact association still needs to be determined. However, our results show a low prognostic value of D-dimer levels on ICU referral when taken alone. In the same line, this finding could be similar to the use of D-dimer in PE, which has a low predictive performance when using absolute values and needs an age-adjusted cutoff. Interestingly, we previously observed that the of D-dimer level <500 ng/mL associated with female gender and absence of pneumonia at CT scan as potential exclusion criteria for COVID-19 diagnosis (17). Increase in D-dimer level and its evolution is probably a better reflect of COVID-19-associated coagulopathy involvement and progression that might help to choose between a prophylactic or a therapeutic anticoagulation strategy. This hypothesis needs to be confirmed in ongoing prospective randomized clinical trials.

The major finding of the present study is the excellent prognostic value of Hs-cTnI level to predict respiratory worsening, ICU referral, and mortality. It is important to note that the proposed Hs-cTnI cutoff is lower than the thresholds used for myocarditis or myocardial infarction diagnosis (48). The observed elevation of Hs-cTnI may probably be more resulting from microcirculatory damage and myocardial oxygen supply–demand mismatch and supply than a primary pathology of the myocardium, as it was already observed in the case of community-acquired pneumonia (21, 49). In parallel with myocardial inflammation due to SARS-CoV-2 infection, myocardial damage could result from a RV wall tension due to an increased RV afterload with RV dilation and tricuspid valve insufficiency. Yet, the mechanism of troponin release from the myocardium is not fully understood, and various pathophysiological scenarios have been proposed (50). However, the higher the troponin, the higher the risk of ICU referral (51, 52), with no altered LV function probably underlining the burden of endotheliitis and microthrombotic processes in the outcome of patients with COVID-19.

**Figure 2:**
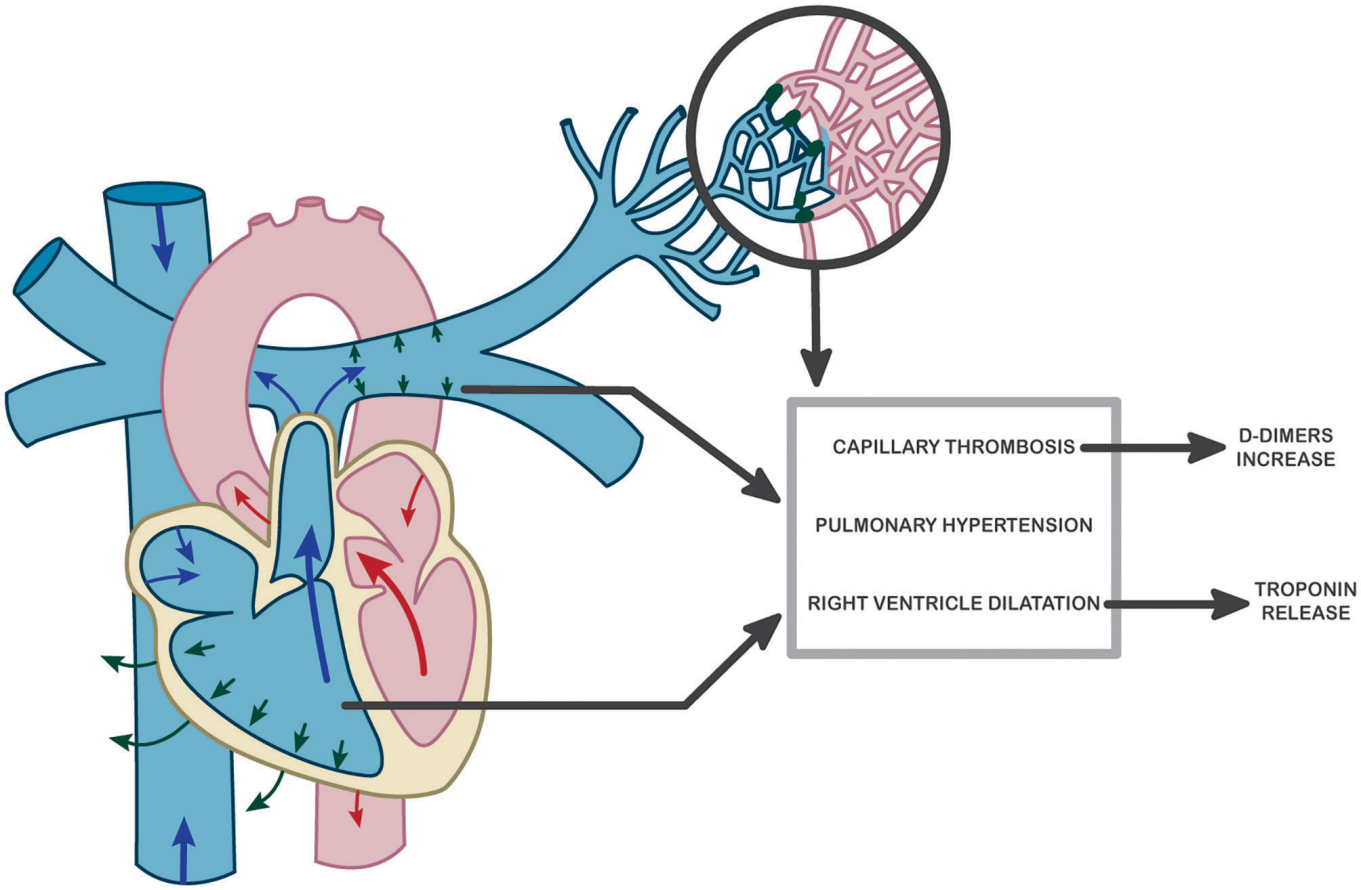
Hypothesis of a potential pathophysiological mechanism explaining pulmonary and cardiac dysfunction in COVID-19 and resulting in troponin and D-dimer increase at admission to the hospital.

In the present study, Hs-cTnI was clearly correlated with sPAP values as well as with RV longitudinal systolic dysfunction. These results suggest that an increase in pulmonary pressures secondary to capillary microthrombosis may be responsible for right ventricular myocardial distress, which leads to troponin release. This hypothesis is also supported by the results of Szekely et al. showing RV dysfunction at the forefront of cardiac damage associated with COVID-19 and correlated with troponin levels (53). Although troponin was the best prognostic marker of patient worsening, D-dimer levels also have a prognostic role, appearing to correspond to a causal process of pulmonary microthrombosis. Thus, we suggest here a kinetic study of cardiopulmonary-induced lesion in COVID-19 as proposed in Figure 2. Pulmonary endothelium forms a key part of the alveolar–capillary unit, providing an interface for efficient gas exchange between the alveolar space and blood cells within lung capillaries. We previously described an early endothelial lesion that drives prognosis and ICU transfer of patients. This thrombo-inflammatory process in pulmonary vessels is probably the main actor of microthrombosis in lung capillaries (reflected by increased D-dimer) driving consequences in right ventricle. Thus, troponin increase is mainly reflecting RV afterload increase.

## Limitations

This is a pilot study on the search for prognostic biomarkers of COVID-19, explaining this single-center work on a limited number of patients. We also do not present troponin kinetics, which were not systematically performed. A longer follow-up will be required to firmly define prognostic biomarkers of COVID-19 severity, and our results should be replicated in a multicenter study. Due to the small simple size and to respect the assumptions required to perform logistic regression, we were authorized to include in the model only these two variables (Hs-cTnI and D-dimer). For this reason, the influence of parameters such as gender or prior anticoagulant treatment (only three patients were treated for atrial fibrillation) could not be reasonably studied in this work. Thus, taking into account all of these limitations, the pathophysiological link between right ventricular afterload, cardiac troponin release, and COVID-19 is first and foremost a hypothesis requiring broader validation.

Despite these limitations, we believe that Hs-cTnI provides important information on the severity of COVID-19, like in pulmonary embolism, even with a cutoff value below the threshold usually used for acute coronary syndrome diagnosis (11.6 pg/mL for women and 19.8 pg/mL for men). Therefore, with the condition that each center adjusts its cutoff according to the intervariability of the method, Hs-cTnI could be considered as a relevant surrogate marker to avoid any delay in COVID-19 patient care and referral to ICU. Despite these limitations, we believe that, like in pulmonary embolism, Hs-cTnI, even at a low value below the threshold usually used (11.6 pg/mL for women and 19.8 pg/mL for men), provides important information on the severity of COVID-19.

In conclusion, it seems consistent to open the way for biomarkers in cardiovascular complications and coagulation disorders in COVID-19 patients at admission to the hospital. Further prospective studies should not only evaluate Hs-cTnI and D-dimer levels utility to predict admission to ICU but also evaluate their prognostic value during follow-up and their relevance in respiratory and thrombotic related disorders.

## Data Availability Statement

The raw data supporting the conclusions of this article will be made available by the authors, without undue reservation.

## Ethics Statement

The studies involving human participants were reviewed and approved by Comité de protection des personnes. The patients/participants provided their written informed consent to participate in this study.

## Author Contributions

DS, TM, and J-LD conceived and supervised the study. GG, J-LA, LK, NG, and BD monitored and analyzed the data. RC analyzed the data and supervised statistical analysis. All authors interpreted the data, drafted and revised the manuscript, and approved the final version.

## Conflict of Interest

The authors declare that the research was conducted in the absence of any commercial or financial relationships that could be construed as a potential conflict of interest.
