# Supplementary material for: Predictive Factor for COVID-19 Worsening: Insights for High-Sensitivity Troponin and D-Dimer and Correlation With Right Ventricular Afterload
Source: Front Med (Lausanne). 2020 Nov 12;7:586307. doi: 10.3389/fmed.2020.586307 (PMC7689153; doi:10.3389/fmed.2020.586307)
Supplement: Supplementary file 1 [file Data_Sheet_1.docx]

**Predictive factor for COVID-19 worsening: insights for high-sensitivity troponin and D-dimers and correlation with right ventricular afterload.**

**Guillaume GOUDOT, Richard CHOCRON et al.**

**Supplementary files**

**Supplementary Table 1: Clinical characteristics of patients for whom cardiac ultrasound has been performed (in conventional hospitalization or intensive care unit).**

|  | **Medicine ward unit patients (n=32)** | **ICU patients (n=40)** | **Total**  **(n=72)** | ***p*-value** |
| --- | --- | --- | --- | --- |
| Age  (year) | 64.5  [51.5 – 72.8] | 63.5  [55.8 – 72.0] | 63.50  [55.0 – 72.0] | 0.914 |
| Male sex  (%) | 68.8 | 80.0 | 75.0 | 0.411 |
| BMI  (kg/m²) | 27.2  [23.6 – 30.5] | 28.2  [26.7 – 33.5] | 27.9  [25.7 – 32.4] | **0.044** |
| **Past medical history** | | | | |
| Hypertension  (%) | 50.0 | 55.0 | 52.8 | 0.853 |
| Diabetes mellitus  (%) | 15.6 | 47.5 | 33.3 | 0.162 |
| Current smoker  (%) | 9.4 | 5.0 | 6.9 | 0.720 |
| Hyperlipidaemia  (%) | 59.4 | 40.0 | 51.4 | 0.162 |
| Coronary artery disease (%) | 9.3 | 22.5 | 16.7 | 0.224 |
| Previous stroke  (%) | 6.2 | 2.5 | 5.6 | 0.653 |
| Previous cancer  (%) | 18.8 | 2.5 | 9.7 | 0.050 |
| **Haemodynamic parameters during echocardiography** | | | | |
| FiO2  (%) | 21.0  [21.0 – 28.0] | 50.0  [43.8 – 100.0] | 35.0  [25.0 – 55.0] | **<0.001** |
| Respiratory rate  (min^–1^) | 22.0  [20.0 – 24.0] | 31.0  [22.8 – 34.8] | 27.0  [20.0 – 34.0] | **0.010** |
| Mean arterial pressure (mmHg) | 90.5  [82.0 – 101.0] | 70.0  [60.5 – 78.00] | 78.0  [67.5 – 92.5] | **<0.001** |
| Heart rate  (min^–1^) | 82.5  [74.0 – 95.0] | 105.0  [85.0 – 116.0] | 89.0  [79.0 – 109.0] | **<0.001** |

BMI : Body mass index. ICU: Intensive care unit.

Results are presented as median and interquartile (25th and 75th).

TTE: transthoracic echocardiographic, ICU: Intensive care unit, PEEP: Positive End-expiratory pressure, MAP: mean arterial pressure, HR: heart rate.

| Blood gas sample, respiratory and haemodynamics collected at the time of TTE in ICU. | |
| --- | --- |
| pH | 7.28 [7.20 – 7.34] |
| PaCO_2_ (mmHg): | 53 [42 – 59] |
| PaO_2_ (mmHg): | 85 [68 – 100] |
| Lactate (mmol/L): | 1.45 [1.10 –2.00] |
| FiO_2_ (%) | 50 [44 – 100] |
| PEEP (cmH_2_O) | 13 [5 – 16] |
| Plateau pressure (cmH_2_O) | 26 [19 – 28] |
| Respiratory system compliance (mL/cmH_2_O) | 36.3 [31.1 – 45.9] |
| MAP (mmHg): | 70 [60 – 78] |
| HR/min: | 105 [85 – 116] |
| Epinephrine (mg /h): | 0.0 [0.0 – 0.9] |

**Supplementary Table 2: Blood gas sample, respiratory and haemodynamics collected at the time of TTE in ICU**

**Supplementary Table 3: Demographic, clinical and biological characteristics of COVID-19 patients according to ultra-sensitivity troponin cutoff at admission.**

|  | **Patients with Hs-cTnI level <9.75 pg/mL (n=27)** | **Patients with Hs-cTnI level >9.75 pg/mL (n=48)** | ***p-value*** |
| --- | --- | --- | --- |
| Male sex – n (%) | 19 (65.5) | 40 (83.3) | 0.131 |
| Age - years, median [IQR] | 55.0  [38.0 – 63.0] | 63.50  [55.0 – 75.0] | 0.010 |
| BMI - kg/m², median [IQR] | 27.2  [26.1 – 31.0] | 27.40  [24.50 – 28.90] | 0.494 |
| Time from illness onset to hospital admission - days | 6.0 [3.0 – 7.0] | 7.0 [4.0 – 9.3] | 0.044 |
| **CV risk factors, n (%)** |  |  |  |
| Hypertension | 10 (34.5) | 24 (50.0) | 0.27 |
| Dyslipidemia | 8 (27.6) | 19 (39.6) | 0.411 |
| Diabetes | 8 (27.6) | 13 (27.1) | 0.178 |
| Sedentary lifestyle | 1 (3.4) | 3 (6.2) | 0.455 |
|  |  |  |  |
| Chronic kidney disease | 1 (3.4) | 6 (12.5) | 0.353 |
|  |  |  |  |
| **Medical history, n (%)** |  |  |  |
| Cancer | 1 (3.4) | 3 (6.2) | 0.995 |
| Coronary heart disease | 0 (0.0) | 6 (12.5) | 0.016 |
| Stroke | 2 (6.9) | 4 (8.3) | 1.000 |
| **Treatments, n (%)** |  |  |  |
| Statins | 3 (10.3) | 14 (29.2) | 0.100 |
| Oral antidiabetic agents | 6 (20.7) | 10 (20.8) | 1.000 |
| Insulin | 2 (6.9) | 5 (10.4) | 0.911 |
| β-blocker | 0 (0.0) | 9 (18.8) | 0.034 |
|  |  |  |  |
| Calcium channel blockers | 3 (10.3) | 11 (22.9) | 0.280 |
| ACEi or ARBs | 3 (10.3) | 21 (43.8) | 0.005 |
| ARBs | 1 (3.4) | 10 (20.8) | 0.021 |
|  |  |  |  |
| Diuretics | 1 (3.4) | 8 (16.7) | 0.167 |
|  |  |  |  |
| Central acting agent | 0 (0.0) | 0 (0.0) | - |
| **Clinical features, n (%)** |  |  |  |
| Fever | 29 (100.0) | 44 (91.7) | 0.286 |
| Headache | 10 (34.5) | 18 (37.5) | 0.427 |
| Cough | 26 (89.7) | 40 (83.3) | 0.19 |
| Productive cough | 2 (6.9) | 3 (6.2) | 1.000 |
| Dyspnea | 16 (55.2) | 37 (77.1) | 0.083 |
| Myalgia | 9 (31.0) | 16 (33.3) | 1.000 |
| Diarrhea | 2 (6.9) | 10 (20.8) | 0.12 |
| Pneumonia at CT-Scan | 22 (75.9) | 45 (93.8) | 0.056 |
| ARDS | 1 (3.4) | 12 (25.0) | 0.033 |
| SpO2 - %, median [IQR] | 93.0 [91.0 – 96.0] | 90.5 [87.0 – 95.0] | 0.018 |
| Respiratory rate - Breathes per min, median [IQR] | 20.0 [17.75 – 24.0] | 21.0 [18.3 – 25.0] | 0.313 |
| Pulse - Beats per min, median [IQR] | 92.0 [82.0 – 103.0] | 90.0 [81.0 – 106.0] | 0.943 |
| **Biological parameters, n (%)** |  |  |  |
| White blood cells - x10^9^ per L, median [IQR] | 5.50  [4.20 – 6.40] | 6.70  [4.95 – 9.70] | 0.026 |
| Haemoglobin - g/L, median [IQR] | 142.0  [127.0 – 149.0] | 130.0  [116.5 – 144.0] | 0.090 |
| Platelet count - x10^9^ per L, median [IQR] | 169.0  [149.0 – 220.0] | 165.0  [123.5– 252.5] | 0.949 |
| Polynuclear neutrophils - x10^9^ per L, median [IQR] | 3.80  [2.78 – 4.86] | 5.33  [3.65 – 7.59] | 0.014 |
| Lymphocytes - x10^9^ per L, median [IQR] | 0.93 [0.81– 1.25] | 0.69 [0.46 – 1.06] | 0.009 |
| Monocytes - x10^9^ per L, median [IQR] | 0.35 [0.30 – 0.53] | 0.37 [0.23 – 0.53] | 0.802 |
| CRP - mg/L, median [IQR] | 65.4  [22.8 – 94.8] | 140.0  [82.3 – 200.0] | <0.001 |
| Plasma creatinine - µmol/L, median [IQR] | 72.50  [62.75 – 82.75] | 101.00  [78.50 – 140.25] | <0.001 |
| PT ratio, median [IQR] | 0.98 [0.94 – 1.10] | 0.88  [0.84 – 0.98] | 0.001 |
| Fibrinogen - g/L, median [IQR] | 5.5  [4.9 – 5.8] | 6.3  [5.0 – 6.9] | 0.011 |
| D-dimers ≥1000 ng/ml - n (%) | 8 (27.6) | 32 (66.7) | 0.002 |
| D-dimers ≥2000 ng/ml - n (%) | 2 (6.9) | 15 (31.2) | 0.031 |
| D-dimers – ng/mL, median [IQR] | 687  [51 – 1127] | 1410.  [957 – 2237] | <0.001 |
| Fibrin monomers - µg/mL, median [IQR] | <7.0 [<7.0 – <7.0] | <7.0 [<7.0 – <7.0] | 0.677 |

### BMI: body mass index; CV: cardiovascular; ACEi : angiotensin conversion enzyme inhibitor; ARB-2: antagonist of angiotensin 2 receptor blocker ARDS: acute respiratory distress syndrome; SpO2: pulse oximetric saturation; IQR: interquartile range. CRP: C-reactive protein; Hs-cTnI: high-sensitivity troponin I.; PT: thromboplastin time.

**Supplementary Table 4: Ultrasound characteristics of patients, performed in conventional hospitalization or intensive care unit.**

|  | **Medicine ward patients (32)** | **ICU patients (40)** | **Total (72)** |
| --- | --- | --- | --- |
| **Left ventricle parameters** | | | |
| (LVEDD (long axis, parasternal view) (mm) | 44.0  [41.9 – 49.6] | 45.0  [41.8 – 50.2] | 44.0  [41.9 – 50.0] |
| LVESD (long axis, parasternal view) (mm) | 29.5  [25.9 – 32.0] | 33.0  [29.5 – 37.7] | 31.0  [26.4 – 37.0] |
| LVEF  (%) | 60.0  [60.0 – 65.0] | 60.0  [53.5 – 65.0] | 60.0  [55.0 – 65.0] |
| E wave (mitral flow)  (cm/s) | 66.0  [56.9 – 76.8] | 75.0  [59.0 – 85.2] | 70.0  [57.5– 83.5] |
| A wave (mitral flow)  (cm/s) | 79.0  [69.5 – 91.0] | 61.4  [54.3 – 71.3] | 69.6  [59.2 – 84.9] |
| Lateral e’ wave  (cm/s) | 11.75  [9.38 – 14.32] | 10.60  [7.81 – 12.95] | 10.90  [8.10 – 14.15] |
| E/A ratio  (no unit) | 0.8  [0.7– 1.1] | 1.2  [0.8 – 1.4] | 0.9  [0.7 – 1.4] |
| E/ lateral e’ ratio  (no unit) | 5.45  [4.72 – 7.60] | 6.85  [5.40– 8.73] | 6.20  [4.93 – 8.28] |
| **Right ventricle parameters** | | | |
| TAPSE  (mm) | 21.8  [17.0 – 26.3] | 20.0  [18.0– 22.6] | 21.0  [17.8 – 23.6] |
| S’ TDI  (cm/s) | 12.5  [11.0 – 15.6] | 13.5  [12.0 – 17.2] | 13.0  [11.5 – 16.0] |
| sPAP  (mmHg) | 24.5  [19.3 – 30.0] | 42.8  [34.3 – 49.2] | 31.6  [25.1 – 43.6] |
| TR Vmax  (m/s) | 2.08  [1.82 – 2.29] | 2.80  [2.34 –3.00] | 2.34  [2.01– 2.88] |
| RV diameter (apical view) (mm) | 36.0  [32.3– 39.0] | 38.6  [35.0 – 45.0] | 37.8  [33.0 – 43.3] |
| RV/LV ratio  (no unit) | 0.8  [0.7– 0.9] | 0.8  [0.7– 0.9] | 0.8  [0.7– 0.9] |

LV: left ventricle; RV: right ventricle; LVEDD: left ventricular end diastolic diameter; Left ventricular end systolic diameter; LVEF: left ventricular ejection fraction; sPAP: systolic pulmonary artery pressure; TAPSE: tricuspid annular plane systolic excursion ;S’ TDI: S’ wave, Tissue Doppler imaging; TR Vmax : Maximal velocity of the tricuspid regurgitation flow.
